# User Reviews of Depression App Features: Sentiment Analysis

**DOI:** 10.2196/17062

**Published:** 2021-12-14

**Authors:** Julien Meyer, Senanu Okuboyejo

**Affiliations:** 1 School of Health Services Management Ted Rogers School of Management Ryerson University Toronto, ON Canada; 2 Department of Computer and Information Science Covenant University Ota Nigeria

**Keywords:** mHealth, depression, app reviews, natural language processing, app features, emotions, use, linguistic inquiry word count, mobile phone

## Abstract

**Background:**

Mental health in general, and depression in particular, remain undertreated conditions. Mobile health (mHealth) apps offer tremendous potential to overcome the barriers to accessing mental health care and millions of depression apps have been installed and used. However, little is known about the effect of these apps on a potentially vulnerable user population and the emotional reactions that they generate, even though emotions are a key component of mental health. App reviews, spontaneously posted by the users on app stores, offer up-to-date insights into the experiences and emotions of this population and are increasingly decisive in influencing mHealth app adoption.

**Objective:**

This study aims to investigate the emotional reactions of depression app users to different app features by systematically analyzing the sentiments expressed in app reviews.

**Methods:**

We extracted 3261 user reviews of depression apps. The 61 corresponding apps were categorized by the features they offered (*psychoeducation*, *medical assessment*, *therapeutic treatment*, *supportive resources*, and *entertainment*). We then produced word clouds by features and analyzed the reviews using the Linguistic Inquiry Word Count 2015 (Pennebaker Conglomerates, Inc), a lexicon-based natural language analytical tool that analyzes the lexicons used and the valence of a text in 4 dimensions (*authenticity*, *clout*, *analytic*, and *tone*). We compared the language patterns associated with the different features of the underlying apps.

**Results:**

The analysis highlighted significant differences in the sentiments expressed for the different features offered. Psychoeducation apps exhibited more *clout* but less authenticity (ie, personal disclosure). *Medical assessment* apps stood out for the strong negative emotions and the relatively negative ratings that they generated. *Therapeutic treatment* app features generated more positive emotions, even though user feedback tended to be less authentic but more analytical (ie, more factual). *Supportive resources* (connecting users to physical services and people) and *entertainment* apps also generated fewer negative emotions and less anxiety.

**Conclusions:**

Developers should be careful in selecting the features they offer in their depression apps. *Medical assessment* features may be riskier as users receive potentially disturbing feedback on their condition and may react with strong negative emotions. In contrast, offering information, contacts, or even games may be safer starting points to engage people with depression at a distance. We highlight the necessity to differentiate how mHealth apps are assessed and vetted based on the features they offer. Methodologically, this study points to novel ways to investigate the impact of mHealth apps and app features on people with mental health issues. mHealth apps exist in a rapidly changing ecosystem that is driven by user satisfaction and adoption decisions. As such, user perceptions are essential and must be monitored to ensure adoption and avoid harm to a fragile population that may not benefit from traditional health care resources.

## Introduction

### Background

Major depressive disorders account for almost 300 million cases worldwide, with a loss of 63 million disability-adjusted life years every year [1]. Effective treatments are frequently unavailable to those with the greatest need [2,3]. Barriers to receiving mental health and behavioral care include transportation problems, time constraints, costs, emotional barriers, and stigma [3]. Young adults, in particular, tend to have a negative opinion of the mental health care system, feel disconnected from its services, and prefer handling their concerns by themselves rather than resorting to mental health care services [4].

Mobile health (mHealth) apps deliver health care through mobile information technologies such as smartphones and offer an opportunity to address these barriers and expand the reach of depression care, especially in areas with limited or no specialists. They offer multiple advantages such as quasi-unlimited capacity, 24/7 availability, equitable access, anonymity, tailored approach, links to other systems, and low cost [5]. Patients also tend to prefer psychological treatment to medication [4]. As a result, mHealth apps have been installed millions of times [6]. mHealth apps can address a variety of needs and researchers have identified 6 categories of features offered by depression apps, as summarized in Textbox 1 [6,7].

**Definition of depression apps features.**

Psycho-education

- Educate, train or inform users through books, guides, news, journal articles, commentaries or opinions, tips, and lessons.

Medical assessment

- Allow users to screen, diagnose, assess risks, assess self, determine treatment.

Symptom management

- Allow users to track symptoms, gather history, including physical health data and provide useful, comprehensible output.

Therapeutic treatment

- Prescribe solutions to improve the condition (therapeutic or not). Includes relaxation, hypnosis, mindfulness exercises, meditation, spiritual or faith-based solutions.

Supportive resources

- Provide referrals for help, connects users with support, for example, emotional and social support, treatment interventions for acute or chronic use, etc.

Entertainment

- Serve recreational purposes, such as quotes, dark humor, wall papers and games.

Multifeature

- Offer 2 or more of the above features.

Evidence suggests that mHealth apps can be effective for various mental health disorders, including depression [8-12]. Nevertheless, evidence remains scarce and incomplete. First, there are a limited number of studies and participants involved [9] and they rarely focus on the benefits and usefulness of specific features [6,7]. Second, the rapid evolution of these apps implies that the investment in clinical evaluations of specific apps may be short-lived; for instance, a study showed that 50% of the depression apps available were different after 130 days, with a new app for depression being available every 3 days [13].

Moreover, the clinical relevance seems to be unrelated to adoption [13]. App developers indicate that app stores are increasingly their favorite distribution channel, over health care providers, in line with the evolution of user decision patterns toward increased patient empowerment [14]. Young users, in particular, often openly reject being told what app to use [15]. Thus, adoption of mHealth apps is increasingly driven by patient attitudes about and experiences with the apps than by clinical evidence and professionals’ prescriptions. Therefore, to better address user needs and improve the adoption and use of adequate apps, we need to better understand the experiences, behaviors, and attitudes of the users of these mHealth apps [16].

App reviews are one key source of information on apps. App users are invited by app stores to rate the apps they have installed and write free text reviews about them. Users willingly and spontaneously contribute to these reviews. Although only a fraction of users choose to do so, these reviews aggregate into a large data set of mostly authentic and publicly available data on user experience with depression apps.

These app reviews are important because of several reasons. First, app reviews can help identify bugs, user requirements, feature requests, user experience with specific app features [17,18] and whether the existing features meet user expectations [19]. Consumer knowledge also reflects different types of knowledge that may be expressed in the reviews, such as knowledge of attributes, the topic, or the buying process [20].

Second, user reviews inform us about the experiences and mental states of the users. For instance, the choice of words in web-based blogs by people with mental health issues helped identify young adults’ suspicions toward the mental health system [4].

Finally, user reviews influence the prospective users’ decision to choose a health care service or not [21]. Specifically, the number of user reviews and user ratings of mental health apps influence adoption [22] and the expression of emotions in user reviews, notably negative emotions, influences how people interpret the reviews [23].

As the scope of human endeavors supported by technology continues to broaden and become more intimate, emotions and values tend to play an increasing role in explaining the adoption and use of a technology [24,25]. Users’ emotional reactions can reflect their assessment of an app, their future propensity to use them, and also reflect some of the impacts of the apps on the users’ condition and behavior.

### Objectives

Considering the importance of user emotions and user reviews in understanding user decisions, this study investigates the emotional reactions expressed in user reviews of depression health apps and analyzes how they relate to app features.

## Methods

### Data Collection

**Figure 1:**
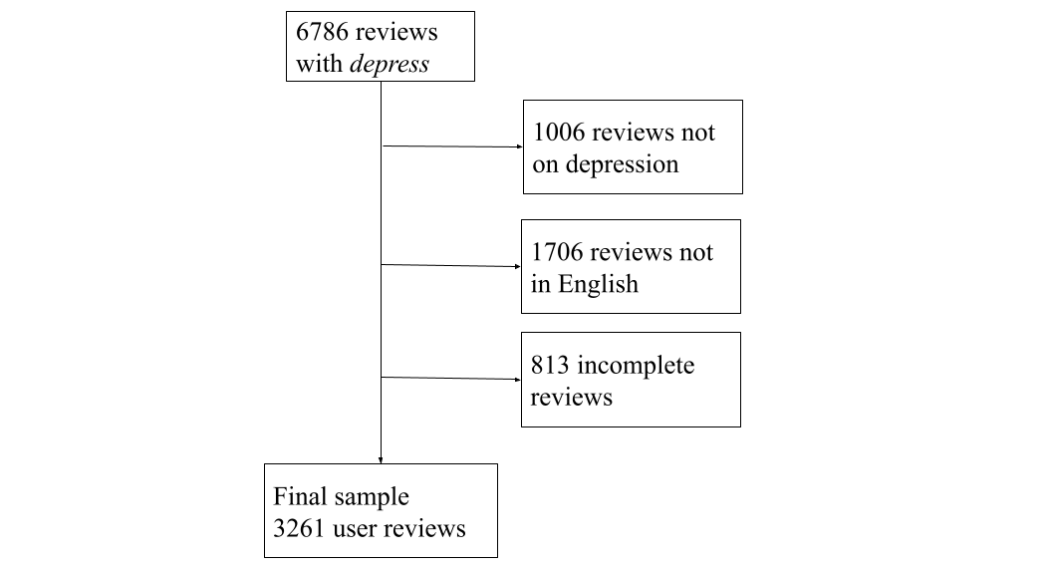
Diagram of app reviews selection process.

Depression apps and their reviews were scraped from Google Play Store and Apple App Store through 42Matters, a third-party application programming interface provider, based on all apps worldwide that included the root *depress-* either in the title or in the description. The search was conducted in March 2018. The data set was then cleaned manually by a researcher to remove non–English-language app reviews or apps and apps unrelated to the mental condition (eg, depression used as a geological term) and apps with missing data (Figure 1). Screening of the app reviews resulted in a final data set containing 3261 app reviews associated with 61 apps.

Each app was categorized using functional categories defined in other studies on depression apps [6,7]. For each app, a researcher read the description of the app, installed the app if necessary, and coded the feature or features provided by the app (refer to Textbox 1 for the coding scheme).

### Statistical Analysis

In app reviews, users report and document their experiences in an unstructured and nonmethodical manner. The volume, variety, velocity, and veracity of user reviews also contributed to making them difficult to analyze.

As a first step, we created a word cloud of the content of the app reviews by functional category. This provided an intuitive, unmediated idea of some of the themes and concepts reflected in the reviews. We used the statistical and data management software R to remove common English stop words (plus *depression,* *depressed* and *app*) and used the 50 most frequently used words for each group of app reviews (refer to Multimedia Appendix 1 for the list and frequencies of the most frequently used words by feature).

We then used a text-mining tool to analyze the content of the reviews. Natural language processing involves techniques to analyze large data sets of natural (ie, not codified) language and has been critical for understanding consumer attributes and behaviors [26,27]. Sentiment analysis is a subset of natural language processing that investigates thoughts, emotional reactions, and feelings regarding a specific subject or topic or simply identifies the overall polarity of a topic [21]. It operates by extracting and retrieving information from unstructured raw text and extracting words or grammatical patterns that reflect emotions or thought processes. In health care, these analytical methods have been used, for instance, to interpret textual information about patient experience [21,28] or patient satisfaction [29].

App reviews were analyzed using Linguistic Inquiry Word Count (LIWC) 2015 (Pennebaker Conglomerates Inc). LIWC is a well-established application that analyzes natural language text segments and counts the frequency of words reflecting different emotions, thinking styles, social concerns, and other dimensions [30-32]. LIWC is a lexicon-based approach to semantic analysis, which is based on a predefined dictionary. Although LIWC was not specifically developed to investigate app reviews, lexicon-based approaches to sentiment analysis of consumer reviews do not significantly differ based on the context being analyzed [33]. LIWC has been used in prior studies to extract depression-related linguistic cues from web-based forums [31] and analyze mobile app reviews [19,34].

LIWC codifies more than 92 different aspects of language. It assesses the valence of a text in 4 dimensions (*authenticity*, *clout*, *analytic*, *tone*) by analyzing the linguistic style. Authenticity measures the presence of features associated with true and false stories [32,35]. False stories, for instance, tend to use more motion words and more negative emotion words but fewer first-person pronouns [35]. LIWC then provides a rating from 0 to 100, 50 being neutral. For example, the following review was scored 99 on authenticity, 73.64 on (emotional) tone, but only 1 on *analytic* and *clout*, reflecting that the quote talked more about the user’s experience than about the app:

I already knew this but now I can physically see that I am and I can’t even tell my own parents wow. I don’t know how to get better I really don’t and it said I have server depression.

In contrast, the following review was rated 64.27 on *analytic*, 98.93 on *clout*, reflecting how the user was analyzing the features and trying to influence the designers but not saying much about his or her experience with the app:

Would Love to have Transparent Effect! And an idea for you, make one with Motivational thoughts and people would go CRAZY and Install your App.

LIWC also measures the frequency of certain lexicons, such as *money*, *home*, *you* or *adverb*, (not only these exact words but any words related to the theme). Categories were rated from 0 to 100 to reflect the valence in the linguistic feature, 0 indicating complete absence and 100 indicating that the fragment fully reflected the category. Owing to the purpose of the apps, the codes may reflect the user’s state of mind or the feature being assessed. “Best anxiety tool out there” and “It’s OK but too confusing” were both rated at 20 on anxiety but the first one reflected an analytical stance on anxiety while the other reflected the state of mind of the user. Thus, both meanings were included in the values and could not be disentangled by LIWC.

We coded the app reviews with LIWC, meaning that the complete app reviews were analyzed and rated rather than the individual sentences. Using R, we then performed 2-tailed *t* tests on the relevant dimensions to measure whether there were significant differences between the reviews associated with 1 feature and the depression app reviews overall. We focused on the 4 summary language variables (*analytic*, *clout*, *authenticity*, and *emotional tone*) [32]. Owing to its importance, instead of reporting the emotional tone directly, we reported its subcomponents *positive emotion* and *negative emotion* which have been associated with app adoption [3]. We also added *anxiety*, a subcomponent of *negative emotion*, which was directly related to depression. *Negative emotion*, *positive emotion*, and *anxiety* are lexicon dimensions that reflect frequency rather than valence. Therefore, their values were typically lower than those of the dimensions. Textbox 2 defines the variables that were retained.

**Selected Linguistic Inquiry Word Count dimensions and their definitions.**

Analytic (analytical thinking)

- Degree to which people use words that suggest formal, logical, and hierarchical thinking patterns. People low in analytical thinking tend to write and think using language that is more narrative, focusing on the here-and-now and personal experiences.

Clout

- Relative social status, confidence, or leadership skill that people display through their writing or talking.

Authenticity

- When people reveal themselves in an authentic or honest manner, they are more personal, humble, and vulnerable.

Tone (emotional tone)

- Includes both positive and negative emotion dimensions; the higher the number, the more positive the tone. Ratings below 50 suggest a more negative emotional tone. It was broken down into:

Positive emotion

- The more that people use positive emotion words, the more optimistic they tend to be. If you feel good about yourself, you are more likely to see the world in a positive way.

Negative emotion

- Use of negative emotion words is weakly linked to people’s ratings of anxiety or even neurotic. People who have had a bad day are more likely to see the world through negatively-tinted glasses. Words denoting anxiety (worried, fearful...) are a subset of negative emotion.

Finally, we illustrated the analysis with samples of complete reviews to connect both the word clouds and the language variables with actual uses of the words, as the anecdotal context facilitated the understanding of the analytical process.

## Results

### App Statistics

First, we present basic descriptive statistics on the number of reviews by year of publication (Table 1) and by word count (Table 2).

**Table 1:** Number of reviews by year of publication.

Year of review	Reviews, n
2012	4
2013	5
2014	18
2015	122
2016	138
2017	1760
2018	877
2019	337

**Table 2:** Number of reviews by word count.

Word count	Reviews, n
<5	645
6 to 10	1251
11 to 20	807
21 to 50	868
50 to 100	292
>100	43

Second, we analyzed the number of app reviews by category and the average review rating out of 5 stars (Table 3). No app that offered the feature of *symptom management* exclusively was identified, and therefore, that category was excluded from the remainder of the analysis. The app ratings for *psychoeducation, therapeutic treatment*, and *multifeature* apps were slightly but significantly above average, while those for *medical assessment* apps were significantly below average. The average character count was 134, which was slightly above the average of 117 observed in app reviews in general [36].

**Table 3:** App count, installation, and reviews by functional category.

App category	Exclusive feature app count (total apps with feature; N=61), n (%)	App review count (N=3261), n (%)	Rating, mean (SD)	*P* value^a^	Review length in words, mean (SD)
*Psychoeducation* ^b^	17 (27.86)	259 (7.94)	4.2 (1.3)	.06	18.9 (18.2)
*Medical assessment*	12 (19.67)	556 (17.05)	4.0 (1.4)	<.001	14.8 (15.7)
*Symptom management*	0 (0)	0 (0)	N/A^c^	N/A	N/A
*Therapeutic treatment*	9 (14.75)	293 (8.98)	4.3 (1.3)	.69	18.1 (19.7)
*Supportive resources*	4 (6.56)	138 (4.23)	4.2 (1.4)	.39	20 (24.0)
*Entertainment*	7 (11.47)	353 (10.82)	4.4 (1.2)	.04	14.9 (14.9)
*Multifeature*	12 (19.67)	1662 (50.96)	4.4 (1.0)	<.001	25.8 (27.9)

^a^Welch 2-sample *t* test between all reviews and each category.

^b^References to these categories are italicized in the text.

^c^N/A: not applicable.

### Word Clouds

Third, we report the word clouds of app reviews under each category in Multimedia Appendices 2-7. The word clouds represent the most common terms used in the app reviews. The more frequent a word, the bigger and more central its representation in the cloud. Generic words like *help* or *like/love* appeared across categories; also, category-specific words emerged, like *test* or *result* for *medical assessment* apps, *game* or *quotes* for *entertainment* apps, *people* or *chat* for *supportive resources* apps and *journal* or *meditation* for *therapeutic treatment* apps. However, some less predictable words also appeared and provided hints about the focus of the users. For instance, *severe* (ie, severe depression) appeared specifically at the top in the list of words for *medical assessment* apps or *inspire* in the list for *entertainment* apps, which included *quotes* apps.

### Sentiment Analysis of User Reviews

**Figure 2:**
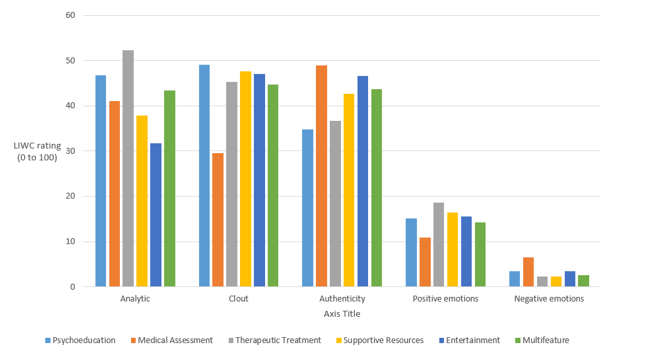
Key sentiment dimensions by single-feature app category. LIWC: Linguistic Inquiry Word Count.

The LIWC sentiment analysis by feature is reported in Table 4 and Figure 2. The *P* value of the *t* test compares feature-specific reviews with other app reviews. For instance, the *analytical* score of 52.2 for *therapeutic treatment* versus an average score of 43 (SD 36.3) for all reviews has *P*<.001, meaning that it is significantly above average.

**Table 4:** Key sentiment dimensions by category^a^.

App category	Analytic rating		Clout rating		Authenticity rating	
	Value, mean (SD)	*P* value	Value, mean (SD)	*P* value	Value, mean (SD)	*P* value
*Psychoeducation* ^b^	46.7 (36.4)	.08	49.2 (33.4)	.001	34.8 (36.6)	<.001
*Medical assessment*	41 (38.1)	.18	29.5 (29.5)	<.001	48.9 (40.8)	.02
*Therapeutic treatment*	52.2 (36.3)	<.001	45.3 (31.2)	.15	36.8 (38.7)	<.001
*Supportive resources*	37.9 (35.0)	.08	47.6 (34.0)	.09	42.6 (40,2)	.43
*Entertainment*	31.8 (34.7)	<.001	47 (34.0)	.01	46.7 (40.0)	.50
*Multifeature*	44.2 (35.6)	.62	44.5 (32.5)	.01	47.15 (39.5)	.11
All reviews	43 (36.3)	N/A^c^	42.8 (32.8)	N/A	45.3 (39.7)	N/A

^a^Welch 2-sample *t* test between all reviews and each category.

^b^References to these categories are italicized in the text.

^c^N/A: not applicable.

*Psychoeducation* app reviews were significantly higher than the average on *clout*, but were less *authentic* (34.8), suggesting that the reviews were more focused on influencing others than on sharing personal experiences and that the users were more confident. This was illustrated by reviews such as the following:

No help at all. Lots of information that is easily available from a single Internet search.

I found this helpful but it needs more information for another star, specially I felt that this app has no information about recovery & medical advice (verified doctors).

*Medical assessment* app reviews had less *clout* (29.5) and more *authenticity* (48.9), but also significantly more negative emotions (6.6). This was illustrated by reviews such as, “Kinda lame ask general questions not really a benefit all ready knew answer” *or* “Only 20% of it was true about me in my test.” Others leaned toward personal disclosure:

I got 25. I am 13 and i literally don’t want my life anymore! I wonder how have i not suicided yet!

*Therapeutic treatment* app reviews were more *analytical* (52.2) and less *authentic* (36.8), suggesting that the reviews were more focused on the actual functions. This was illustrated by reviews such as the following:

Brilliant app I particularly liked the progressive explanation of the cognitive distortions and how to address each accordingly.

So helpful and easily accessible. You can pull the app out whenever you need it.

*Supportive resources* app reviews exhibited less negative emotions (2.3) and anxiety (0.06) than the average. *Supportive resources* reviews focused on the process of connecting to other people as illustrated by, “This a great app to connect with people dealing with similar situations!” or on the app features, illustrated as follows:

I have been a member for a while now, and I love we got a mobile app. Wish we could take chats on it though. But awesome non the less.

*Entertainment* app reviews exhibited feedback that went beyond entertainment concerns, such as the following*:*

This game is so amazing, I’ve been struggling with depression and self harm since I was 9, this game had such an emotional impact on me, I hope more people discover this game soon, yeah, its pretty laggy, but I think the over all message equals it out.

Moreover, some were very negative, such as the following:

As a clinical counselor I would say this app is likely to lead suffers down a dark path.

## Discussion

### Principal Findings

Findings pointed to differences in the emotional experiences of users based on the app features.

*Medical assessment* apps specifically received highly negative reviews. Their app ratings and positive emotions were significantly lower than the average of the depression app reviews, while the negative emotions were higher than the average. A possible explanation is that unlike other categories, *medical assessment* apps provide users with feedback and insights into their own personal conditions and whether they are depressed or not. Research suggests that people who disagree with personal feedback may respond with distress and exhibit strong and long-lasting feelings [37]. These emotions may translate into resentment and negative reactions against the quality and the validity of the app. The high level of authenticity also suggests that the users reveal more about themselves. For developers, this suggests the need for caution before introducing *medical assessment* features in their apps, as they may antagonize their users and possibly distress them. It also raises the question of the impact of medical assessment on users. Distress caused by the outcome may lead the users to seek expert opinion for confirmation or disconfirmation, but it may also lead them to draw negative conclusions about what to expect from medical professionals.

In contrast, *supportive resources* apps generate fewer negative emotions. As these apps mostly connect the users to other people, the emotional response may focus more on the people or the services connected to them than on the apps themselves. *Therapeutic treatment* apps generate more positive emotions and anxiety but are also much more analytical, possibly because they focus on the users’ attention on their actions to mitigate their condition. This suggests that both are safer features for developers to offer, at least to begin with. To a certain extent, *psychoeducation* apps also generate more positive reactions (although not significantly). Their low authenticity level can be explained by the impersonal informational and educational dimensions and it also makes them less risky to implement, to the extent that they do not mislead the users with incorrect information. Higher clout level is associated with higher confidence and social status. Users of psychoeducation apps may require sufficient self-confidence to believe that simply getting access to information is sufficient than more prescriptive features. Thus, these apps may cater to a different, more autonomous population.

*Entertainment* apps offer an ambivalent picture. They insignificantly generate more negative emotions, but their users also express significantly less anxiety. Entertainment could be an alternative way to engage people with depression who are anxious about dealing directly with their condition. This confirms findings from prior research obtained from focus groups, suggesting that people with mental health issues, especially male adolescents, value entertainment features in mental health management apps [15].

### Limitations

This study has limitations. The app marketplace is continuously evolving. The study was based solely on the information available in Google Play Store and Apple App Store. This information is subject to the inclusion criteria put in place by the app stores and the developers. The authenticity of the reviews included in this study was not validated, and the issue of illegitimate or fraudulent reviews is widespread [38]. These carry special risks regarding mental health apps, as they could lead the users to make decisions that may be detrimental to their health. User socio-demographic information was unavailable, even though this is a standard practice with recent studies involving sentiment analysis of app reviews [17,39,40].

Moreover, only the users who posted reviews of the apps were represented, which limits the sample’s representativeness of the population of mental health app users. Reviewers were willing to publicly associate their usernames with a depression app, which many users may be reluctant to do, considering the stigma associated with mental disorders. In addition, even though the apps mostly cater to a population with depression, we do not know whether the reviewers are people diagnosed with clinical depression. Future research may try to replicate these findings by actively selecting respondents with depression and asking them to review apps. Such results can then be compared with those from this study or those from the app stores.

Finally, an assumption of this study is that the reviews can be generalized to the app feature, but they may reflect the idiosyncrasies of the reviewed apps (eg, bugs or ill-designed apps), as the number of apps in each category varies from 4 to 20.

### Comparison With Prior Work

This study contributes to the literature in multiple ways.

In addition to the studies that describe cognitive processes such as satisfaction or confirmation of expectations in mHealth app users [19], our findings suggest that depression apps also generate strong emotions. Emotions form a key element of mental health conditions and access to mental health care [3] and should be of concern to researchers and developers interested in improving the apps.

Depression app use is a health care behavior practiced by a large population with potentially serious mental health conditions [6]. The people routinely use depression apps to access information and assess, track, and manage their condition. Ultimately, they draw conclusions about their condition and take action (or maybe more problematically, do not take necessary action) based on feedback from these apps which could have critical impacts on their mental health if continued without clinical supervision. A major concern of researchers and clinicians regarding depression apps is the clinical validity of these interventions. Few of these apps are rigorously and clinically validated [5,12], and despite efforts to provide clinical evidence, the rapidly changing app environment and user behaviors do not suggest that use will be dominated by clinically validated apps in the near future [5]. Future studies could compare the user reviews of validated apps with those of nonvalidated apps.

Installations of depression apps vastly exceed the number of people accessing mental health care services, and therefore do not compete with traditional care as much as with not accessing care at all [6]. They are typically used as stand-alone self-help programs that are either poorly integrated or entirely not integrated with the continuum of care. How they fit in this continuum is a question by itself. Depression app features include clinically validated, inspired by sound research, alternative unproven approaches, or games with minimal or no clinical claims, several of which can be found in the same app. This indicates unclear differences between the apps used. As such, the use of mental health apps is of interest, both as a clinical intervention and as a common behavior performed by people with mental health conditions.

The reviews of entertainment apps, for instance, suggest that a nonclinical approach may provide help and relief to people with depression, which could lead them to acknowledge their condition, gain confidence in the value of external support, and seek other features in the apps. Thus, entertainment apps may be a stepping stone that does not require the users to recognize their condition and their need for help, considering the stigma and emotional barriers associated with it. They could then serve as a gateway to recognizing the value and seeking professional care for people with serious mental health issues. In contrast, apps may act as a deterrent, either because the users feel that the apps are sufficient or even better than traditional care or because bad app experiences, such as an early and disturbing virtual depression assessment, would cause skepticism toward the value of medical expertise. This requires research on the pathways that patients follow between using depression apps and accessing traditional health care services. In line with approaches that follow up web-based health behaviors of specific groups of people [41], future research could longitudinally follow up depression app user behaviors and decision-making processes to identify these pathways. Such behavior patterns may help tailor apps to diverse populations.

Our findings also highlight the need to discriminate mHealth app use based on the features offered. Typically, researchers categorize apps according to the disease or condition addressed by the apps [6,7]. The features offered by the apps are important. Providing information about a condition and helping the users track their symptoms every day are very different services and our findings show that users react differently to them in their emotions and satisfaction. This suggests breaking down the study of mHealth apps based on the features they offer. This could come either in addition or as an alternative to studying mHealth apps based on the mental health condition that they address. Two mHealth apps that provide the same features for different diseases may have more in common than 2 apps that provide different features for the same disease. This study illustrates a novel way to investigate user beliefs and behaviors toward specific features. Although substantial efforts have been made to extract isolated reviews that specifically mention a feature [17-19], the size of the app ecosystem can make it possible to isolate apps that offer a single feature, thereby capturing reactions of the users who may not specifically mention the feature under consideration.

Sentiment analysis answers the need to rely on reactive vetting tools for mHealth apps (what Olff [5] refers to as *postmarketing surveillance*) in complement to randomized controlled trials. Researchers are increasingly recognizing the value of mining patient-generated web-based content and feedback [21] and this study is a step toward exploiting the potential of natural language content generated virtually by people with mental health disorders. Sentiment analysis of these data can help refine our understanding of how the users behave and react emotionally outside of clinical settings. How individuals communicate, what activities they engage in, and what language they use are potential indicators of mental health; users’ mental health conditions, such as depression, may reveal patterns of web-based behaviors through Twitter feeds [16]. Further studies could assess the extent to which app reviewers fit into this pattern by comparing them with other app reviewers. Word clouds complement the insights provided by sentiment analysis. They provide an unmediated representation of the words and lexicons used by the reviewers. In addition to the emotions that are conveyed, we can see that the users focus on looking for *help* on whether they *like* or *love* *good* apps. We can deduce the typical focus of their reviews, such as *tests* and *results*, for *medical assessment*. They provide face value and a topical complement to sentiment analysis. Other language analysis tools such as Latent Dirichlet Allocation or Structural Topic Modelling could also be used to provide further insights into the app reviews.

One of the major appealing features of depression apps is that they allow people to circumvent the stigma associated with mental health issues and access services privately. Beyond the privacy-conscious population, our findings suggest that many users are willing to publicly share their personal and intimate experiences about depression on public outlets such as app stores. This source of data can be used to improve individual apps, understand general patterns of use, and learn about the beliefs, behaviors, and emotions of patients. This is also a cautious reminder that the users may not realize that they are not just talking to the community of depression app users but are making a public statement through both a personal and public Google or Apple account that can be viewed by the broader community, including people to whom the users may not want their condition to be revealed. Further research is needed to investigate the extent to which the app reviewers are aware of what they are disclosing and to whom.

### Conclusions

This study broadens our understanding of depression app use and user emotions and refines our knowledge of user experience based on the app features used. Users react with observably different emotions and sentiments depending on the features offered by the depression apps. This has implications for clinicians to better orient their patients to the proper apps and for developers to improve their design and handle the delicate and intimate aspects of a vulnerable population. It is also useful for users to better understand the risks and benefits of using mental health apps and for researchers to broaden their understanding of virtual behaviors of people with mental health.

Our understanding of the role of smartphones and other personal technologies, both as a cause of and as a solution to mental health disorders is still limited, and we need to broaden the scope of our investigations to include the emotions associated with these new behaviors in new and authentic data sources such as user reviews.

## Appendix

Multimedia Appendix 1 Ranking and frequencies of the most frequent words by category of single-feature apps.

Multimedia Appendix 2 Word cloud of psychoeducation app reviews.

Multimedia Appendix 3 Word cloud of medical assessment app reviews.

Multimedia Appendix 4 Word cloud of therapeutic treatment app reviews.

Multimedia Appendix 5 Word cloud of supportive resources app reviews.

Multimedia Appendix 6 Word cloud of entertainment app reviews.

Multimedia Appendix 7 Word cloud of multifeature app reviews.

## Abbreviations

LIWC: Linguistic Inquiry Word Count
mHealth: mobile health
