# Supplementary material for: User Reviews of Depression App Features: Sentiment Analysis
Source: JMIR Form Res. 2021 Dec 14;5(12):e17062. doi: 10.2196/17062 (PMC8715360; doi:10.2196/17062)
Supplement: Multimedia Appendix 1 [file formative_v5i12e17062_app1.pdf]

Psychoeducation			Medical Assessment		Therapeutic Treatment		Supportive Resources		Entertainment		Multifeature	
Rank	Word	Count	Word	Count	Word	Count	Word	Count	Word	Count	Word	Count
1	helpful	44	test	79	help	81	help	49	love	98	help	774
2	good	41	result	72	great	45	like	33	quot	88	use	338
3	day	37	sever	67	love	40	feel	27	like	43	realli	327
4	like	36	help	65	use	36	love	25	can	43	like	290
5	really	34	accur	64	thank	32	peopl	22	help	40	feel	244
6	help	33	know	58	good	31	great	21	awesom	40	great	236
7	helps	27	got	51	anxieti	29	time	19	read	35	thank	208
8	get	26	good	46	like	27	get	12	make	34	mood	206
9	track	26	get	43	get	23	tri	12	feel	34	good	204
10	great	23	question	43	nice	20	program	12	realli	32	love	196
11	nice	21	like	38	time	19	make	11	great	31	thought	190
12	love	19	realli	38	just	19	even	11	relat	31	day	182
13	one	19	just	35	video	18	one	10	good	30	time	182
14	know	18	say	34	need	18	say	10	life	28	can	174
15	thanks	18	feel	29	much	18	can	10	inspir	28	just	149
16	use	18	now	29	work	17	realli	10	one	26	question	148
17	just	18	thank	27	one	17	know	9	say	23	think	139
18	people	17	time	26	well	16	wallpap	9	wallpap	23	inform	133
19	useful	17	score	26	journal	16	just	9	just	21	work	130
20	best	17	make	24	joke	16	think	9	thing	21	get	128
21	anxiety	16	see	24	tri	15	now	9	thank	19	make	127
22	bad	13	great	23	can	15	life	9	peopl	19	need	124
23	lot	12	love	23	thought	15	someon	9	save	19	way	121
24	mood	12	need	23	mind	15	talk	9	think	18	much	118
25	keep	12	better	22	way	14	day	9	nice	18	one	115
26	much	12	use	22	realli	13	abl	9	give	17	thing	115
27	way	11	want	22	make	12	chat	9	star	17	track	114
28	want	11	can	21	app	12	amaz	8	phone	16	also	104
29	can	11	answer	21	therapist	12	definit	8	game	15	well	102
30	found	11	think	21	understand	12	thank	8	know	15	tool	96
31	time	11	one	20	best	12	download	8	much	15	lot	95
32	someone	11	even	19	medit	12	need	8	time	14	will	95
33	will	10	thing	18	day	12	peer	8	better	14	diari	93
34	information	10	tell	18	phone	11	awesom	7	get	14	give	90
35	also	10	work	18	life	11	word	7	even	13	recommend	90
36	feel	9	dont	18	awesom	11	will	7	will	13	see	90
37	life	9	took	18	counsel	11	way	7	word	13	keep	89
38	helped	9	right	17	sound	10	pay	7	motiv	13	peopl	87
39	see	9	word	17	download	10	wast	7	amaz	12	better	87

40	daily	9		tri	17		music	10		want	6		best	12		even	86
41	days	9		told	16		develop	10		alway	6		day	12		now	76
42	recommend	8		peopl	16		mani	10		bad	6		download	12		anxieti	74
43	basic	8		life	16		start	10		lot	6		sad	12		easi	74
44	even	8		doctor	15		show	10		use	6		ever	12		nice	73
45	quiz	8		bad	14		see	10		anyth	6		idea	11		answer	72
46	says	8		easi	14		chang	9		free	6		right	11		life	71
47	tracking	8		kill	14		person	9		mobil	6		want	10		look	68
48	add	8		look	13		log	9		worth	6		look	10		tri	66
49	application	8		well	13		motiv	9		messag	6		happi	10		know	61
50	therapist	8		will	13		activ	9		understand	5		alot	9		chang	61
