# Supplementary figures and images for: User Reviews of Depression App Features: Sentiment Analysis
Source: JMIR Form Res. 2021 Dec 14;5(12):e17062. doi: 10.2196/17062 (PMC8715360; doi:10.2196/17062)

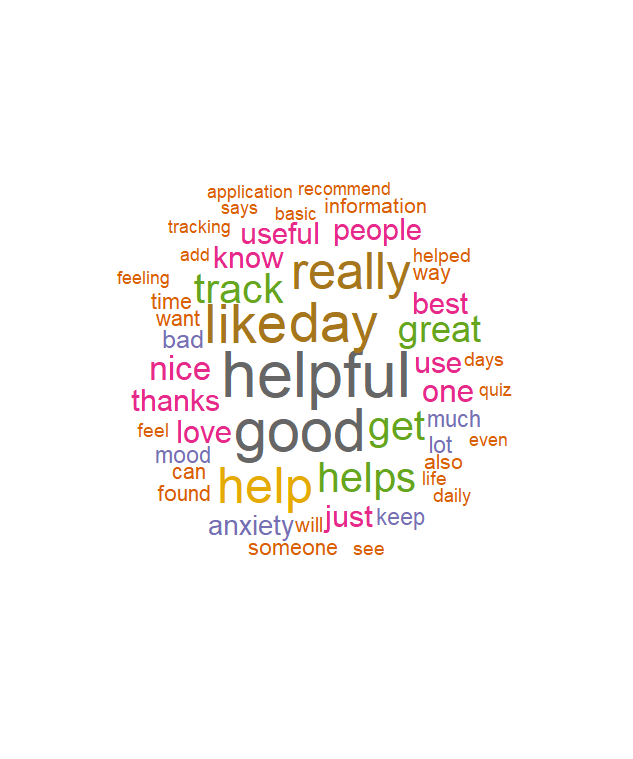

Supplement: Multimedia Appendix 2 [file formative_v5i12e17062_app2.png]

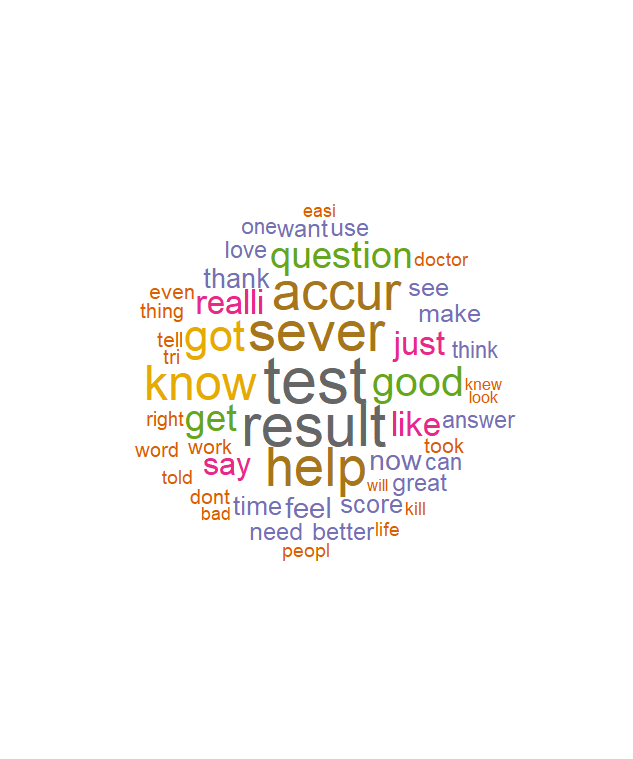

Supplement: Multimedia Appendix 3 [file formative_v5i12e17062_app3.png]

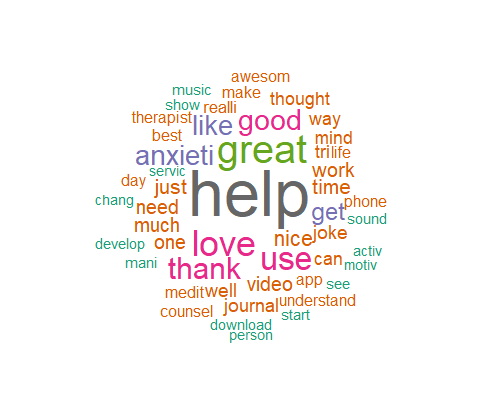

Supplement: Multimedia Appendix 4 [file formative_v5i12e17062_app4.png]

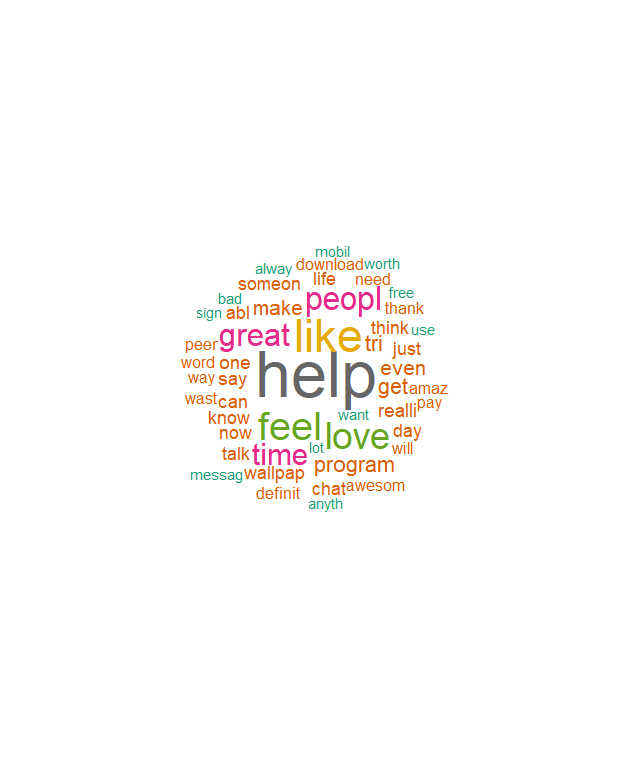

Supplement: Multimedia Appendix 5 [file formative_v5i12e17062_app5.png]

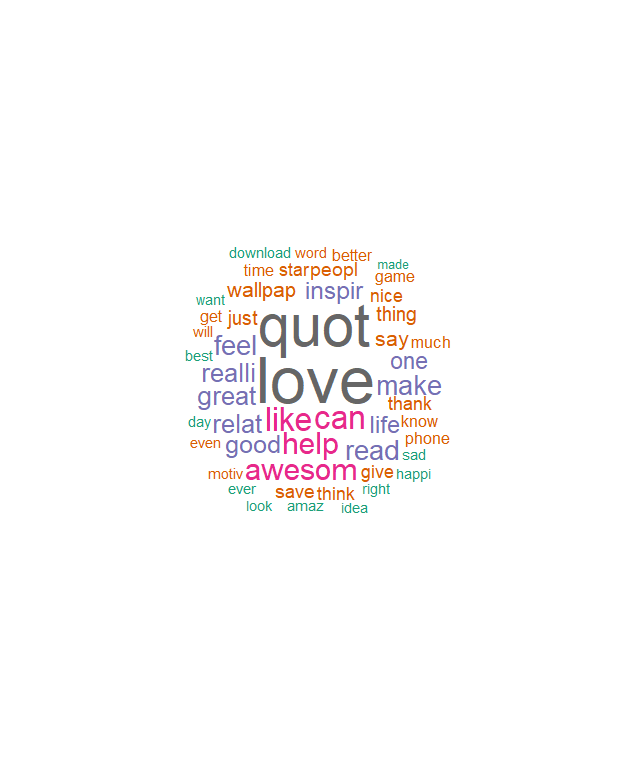

Supplement: Multimedia Appendix 6 [file formative_v5i12e17062_app6.png]

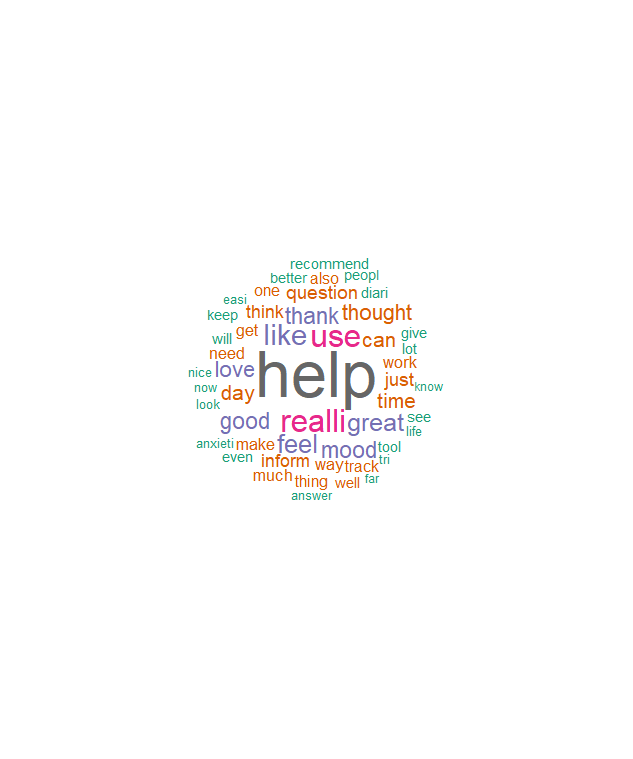

Supplement: Multimedia Appendix 7 [file formative_v5i12e17062_app7.png]
